# Kinase Inhibitors as Underexplored Antiviral Agents

**DOI:** 10.1021/acs.jmedchem.1c00302

**Published:** 2021-05-10

**Authors:** Javier García-Cárceles, Elena Caballero, Carmen Gil, Ana Martínez

**Affiliations:** Centro de Investigaciones Biológicas Margarita Salas (CSIC), Ramiro de Maeztu 9, 28040 Madrid, Spain

## Abstract

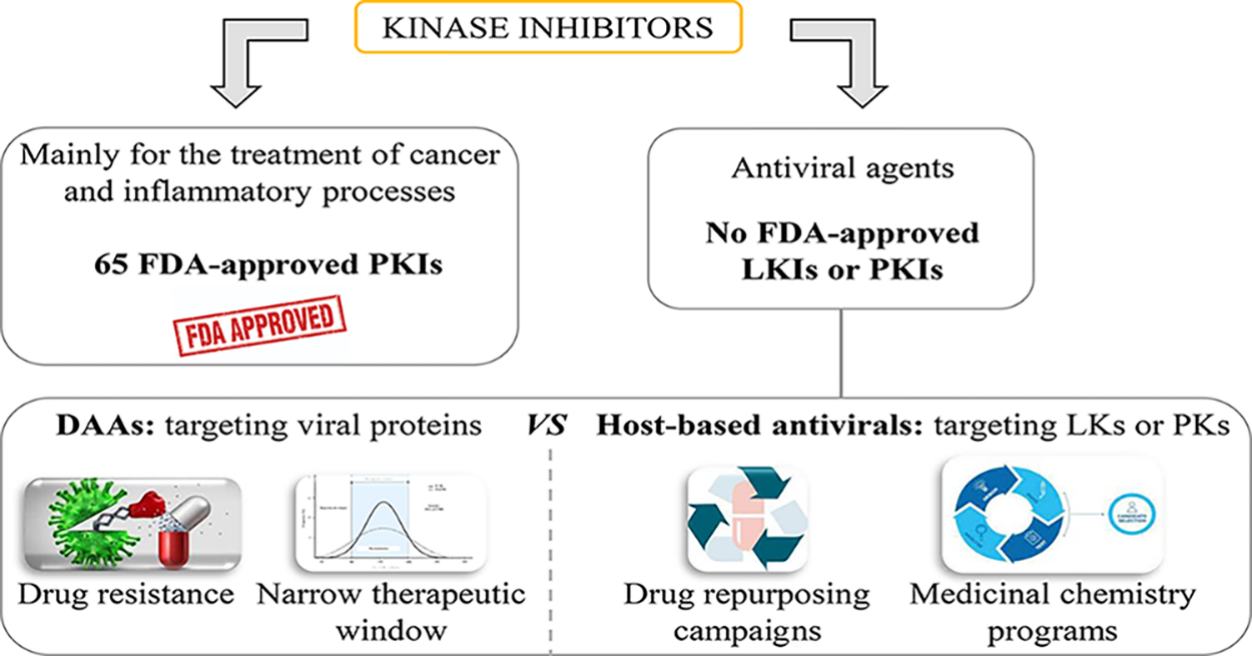

Viral infections are a major health problem; therefore, there is an urgent need for novel therapeutic strategies. Antivirals used to target proteins encoded by the viral genome usually enhance drug resistance generated by the virus. A potential solution may be drugs acting at host-based targets since viruses are dependent on numerous cellular proteins and phosphorylation events that are crucial during their life cycle. Repurposing existing kinase inhibitors as antiviral agents would help in the cost and effectiveness of the process, but this strategy usually does not provide much improvement, and specific medicinal chemistry programs are needed in the field. Anyway, extensive use of FDA-approved kinase inhibitors has been quite useful in deciphering the role of host kinases in viral infection. The present perspective aims to review the state of the art of kinase inhibitors that target viral infections in different development stages.

## 1 Introduction

Kinase enzymes are broadly present in nature, and they participate in a myriad of biological processes. These enzymes catalyze the phosphorylation reaction of a wide variety of substrates (e.g., lipids, carbohydrates, proteins, nucleic acids) through the addition of a phosphate group from the terminal phosphate of adenosine triphosphate (ATP). In particular, protein kinases (PKs) phosphorylate proteins mainly at serine, threonine, or tyrosine residues, as part of post-translational modification events. This produces conformational modifications that lead to functional changes.^1^ Since their discovery by Fischer and Krebs in the late 1950s,^2,3^ the field of research has expanded rapidly. PKs are one of the largest families in eukaryotes. There are 518 kinases encoded by approximately 2% of the human genome,^4^ and they are regulated at many levels, from the phosphorylation state to proteolysis and recycling. PKs are traditionally classified into groups (10) and families (256) based on the primary structure of their catalytic domains.^5^ The main groups of kinases are Ser/Thr kinases (STEs), tyrosine kinases (TKs), tyrosine kinase-like kinases (TKLs), casein kinases (CKs), AGC kinases (named after the protein kinase A, G, and C families), Ca^2+^/calmodulin-dependent protein kinases (CaMKs), CMGC kinases (named after the initials of some members, such as CDKs or MAPKs), and receptor guanylate cyclases (RGCs). These proteins play a key role in mediating signal transduction in cells. They regulate various cellular functions including metabolism, cell cycle regulation, survival, and differentiation.^6^ Mutations and deregulation of PKs are linked to abnormal phenotypes.^7^ Since the first crystal structure of the protein kinase A catalytic domain was solved in 1991,^8^ incredible efforts have been made to understand the structural features that drive the phosphorylation reaction and to provide insights into the substrate binding and selectivity.^9^ According to the KLIFS database,^10^ in 2016 there were more than 2900 crystal structures of the catalytic domains of both human and mouse protein kinases deposited in the Protein Data Bank (PDB).^11^

**Figure 1:**
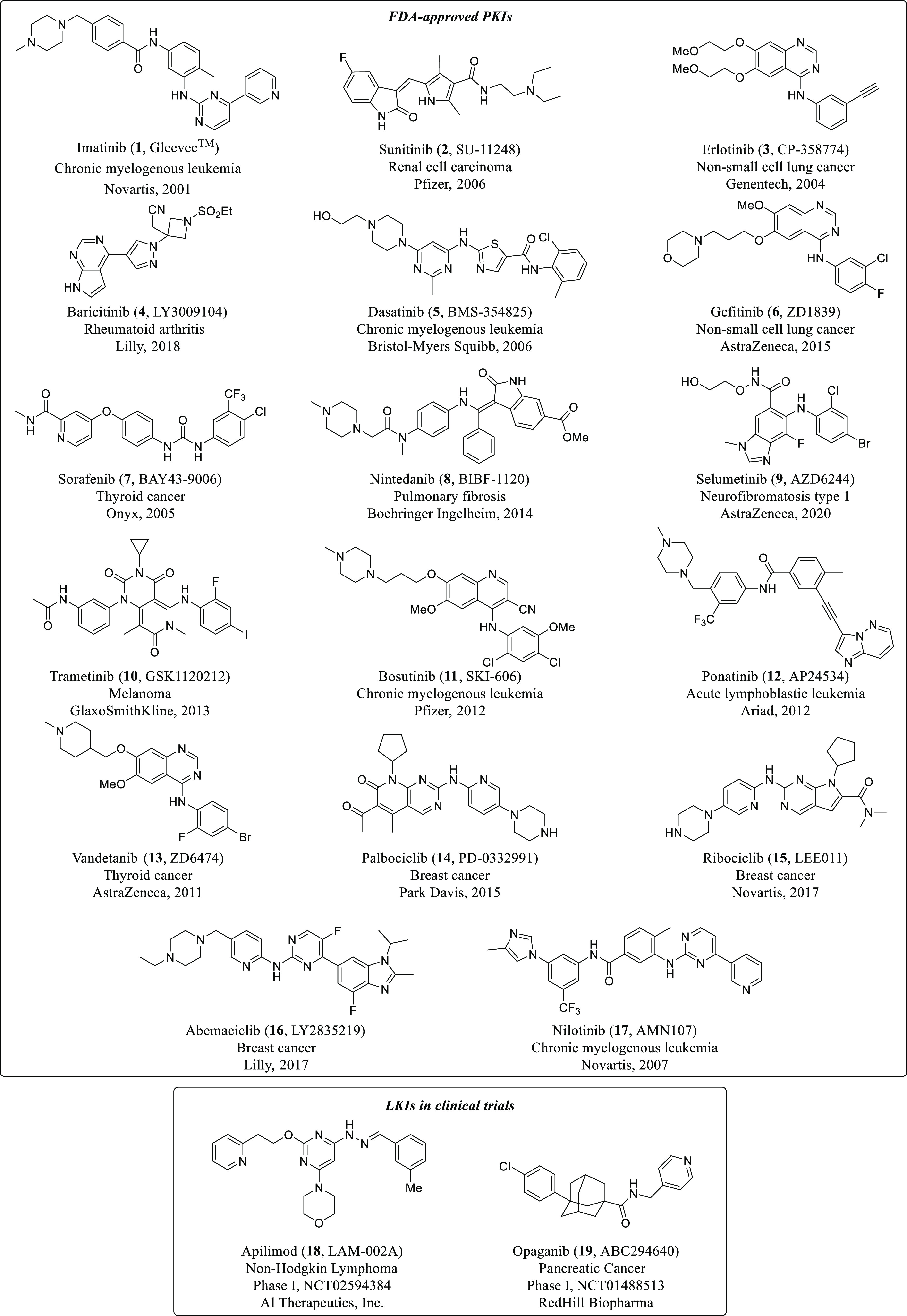
Representative chemical structures of FDA-approved PKIs for cancer or inflammatory processes and antitumoral LKIs with antiviral properties in clinical trials.

Taking into consideration their important physiological role, an extensive number of research programs have been developed, both by academia and industry, toward the identification of ligands (agonists or antagonists) that modulate their activity (activating or blocking, respectively).^12^ In most of the cases, PKs are upregulated in pathological phenotypes, so the search for inhibitors has prevailed. Efforts to develop protein kinase inhibitors (PKIs) as therapeutic agents began as early as the 1980s, when Hidaka disclosed ATP competitive isoquinoline sulfonamide derivatives as inhibitors of various PKs, such as cyclic-nucleotide-dependent protein kinases or protein kinase C (PKC).^13^ However, it was not until 2001 when imatinib^14^ (**1**, Gleevec, Figure 1) was the first PKI approved by the Food and Drug Administration (FDA), targeting Abelson tyrosine kinase (ABL). Since then, FDA approvals have been steadily rising,^15^ with special attention to the treatment of cancer^16^ and inflammatory processes (Figure 1).^17^ Despite this fact, from all of the known human PKs, more than 100 have an unknown function, and 50% of them are largely uncharacterized.^6^ Thus, there is still room for significant breakthrough therapies aimed at the design and development of inhibitors of untargeted PKs. Moreover, recent biological techniques implemented to assess kinase inhibition in living cells offer valuable tools for moving chemical probes to clinical drug candidates.^18^

With respect to lipid kinases (LKs), they phosphorylate lipids in the cell, and they can be classified according to their substrates as diacylglycerol kinases, sphingosine kinases, and phosphatidylinositol kinases. These enzymes make a critical contribution not only to the lipid homeostasis but also to a variety of cellular functions.^19^ LKs have been shown to have an important role in cancer progression. As a consequence, inhibitors of these kinases are being studied as antitumoral agents.^20−22^ Additionally, other relevant functions in diseases related to the cardiovascular system, central nervous system, inflammation, or diabetes have been described.^23−25^ The implication of LKs in these diseases has motivated the development of specific inhibitors to be used as therapeutics.^25−27^ Furthermore, the addition of phosphate groups can change the reactivity and localization of the lipid regulating signal transmission. Identified lipid kinase inhibitors (LKIs) have reached clinical trials (Figure 1), but none of them have been approved for commercialization.

Viral infections are a major health problem in our society; therefore, there is an urgent need for therapeutic strategies to tackle this issue. The most common treatment to avoid infection is vaccination, which is currently a successful approach for the eradication of some diseases (e.g., measles, rubella). Total eradication has been achieved for smallpox and rinderpest viruses.^28^ An alternative method consists of the treatment with antivirals upon infection. Most of these are the so-called direct-acting antivirals (DAAs), and they target proteins encoded by the viral genome.^29^ The main drawbacks of this strategy are the following: (i) drug resistance generated by the virus;^30^ (ii) narrow therapeutic window, making it necessary to develop broad-spectrum antivirals.^31^ An attractive potential solution to overcome these problems may be to aim at host-based targets. Viruses are dependent on a wide variety of host cellular proteins, and phosphorylation events are crucial during their life cycle.^32^ Particularly, a growing body of evidence has shown that viruses hijack several host PKs and LKs at distinct stages of an infection.^33−35^

The development of a new drug is a time-consuming (12–15 years) and expensive (over 1 billion dollars) process.^36^ Thus, repurposing existing kinase inhibitors would help in the cost and the effectiveness of the process because data are already available for selected compounds (toxicity, pharmacokinetics, dosing, etc.), and this helps shorten the clinical pathway. In fact, some repurposing programs have been carried out, and they are rendered promising results.^37−39^ Novel phosphoproteomic programs are a very recent approach to discover kinase inhibitors with antiviral properties.^40,41^ They are based on the analysis and quantification of the phosphorylation pattern upon infection, leading to the identification of the main kinases involved in the process. Therefore, kinases activated during the infection of a virus can be identified as potential therapeutic targets for the development of antivirals.

In the present perspective, we aim to review the state-of-art of kinase inhibitors targeting viral infections, focusing on the most important advances carried out in different kinase families—namely, Numb-associated kinases (NAKs), receptor tyrosine kinases (RTKs), mitogen-activated protein kinases (MAPKs), Src kinases, cyclin-dependent kinases (CDKs), and phosphatidylinositol-3-phosphate-5 kinase (PIKfyve) among others. In order to unravel the mechanism of action of kinase inhibitors as antiviral agents, important attention will be paid to the relationship between infection and kinase regulation.

## 2 Numb-Associated Kinases (NAKs)

The human Numb-associated kinase (NAK) family of Ser/Thr kinases is composed of four known members: adaptor-associated kinase 1 (AAK1), cyclin G-associated kinase (GAK), BMP-2 inducible kinase (BIKE/BMP2K), and serine/threonine kinase 16 (STK16). These kinases are involved in a broad range of cellular functions, and therefore they are related to different diseases such as cancer or Parkinson’s disease.^42^ In fact, there are kinase inhibitors approved for the treatment of different cancers, such as sunitinib^43^ (**2**) or erlotinib (**3**),^44^ and baricitinib^45^ (**4**) for the treatment of rheumatoid arthritis (Figure 1). Also the physiological function of these enzymes has been also related to the regulation of intracellular membrane trafficking. In fact, AAK1 and GAK have key roles in endocytosis,^46^ which has motivated the study of their involvement in processes related to viral infection.

Cells offer a variety of endocytosis, trafficking, and sorting mechanisms that viruses can use to their benefit. In fact, the majority of them need endocytic internalization for penetration and infection. Of the endocytic pathways used by viruses, one of the most commonly used is clathrin-mediated endocytosis.^47^ This process is dependent on the action of the oligomeric clathrin and adaptor protein (AP), and it is widely used by many enveloped RNA viruses. After adhesion to the cell surface and binding to specific cellular receptors, viruses are internalized in clathrin-coated vesicles that are sorted into endosomal compartments. Within endosomes, a drop in pH can induce conformational changes in the viral envelope proteins. This initiates viral fusion with endosomal membranes and the release of the viral RNA into the host cell cytoplasm.^48^ Thus, as clathrin-mediated endocytosis can be considered to be an attractive antiviral target, kinases that regulate clathrin-mediated trafficking can be druggable targets for the development of broad-spectrum antivirals. This is the case of AAK1 and GAK. In fact, by silencing their expression, it has been proved that both kinases are critical for hepatitis C virus (HCV) entry.^49^

The role of AAK1 in receptor-mediated endocytosis is mediated by specific phosphorylation of adaptor protein 2 (AP2), which stimulates the binding to cargo proteins. GAK shares some biological functions with AAK1, and it mediates the binding of clathrin to the plasma membrane and the trans-Golgi network.^50^ Additionally, AAK1 and GAK regulate AP2M1 (μ subunit of the AP2 complex) activity, which is also essential for HCV assembly.^51^ Taken together, these findings show that both enzymes regulate different steps in the virus cycle, entry, and assembly. Thus, they are very useful for the development of novel antiviral strategies. Moreover, the key role of AAK1 and GAK in virus infection has been expanded to other viruses beyond HCV, such as DENV and EBOV.^52^

**Figure 2:**
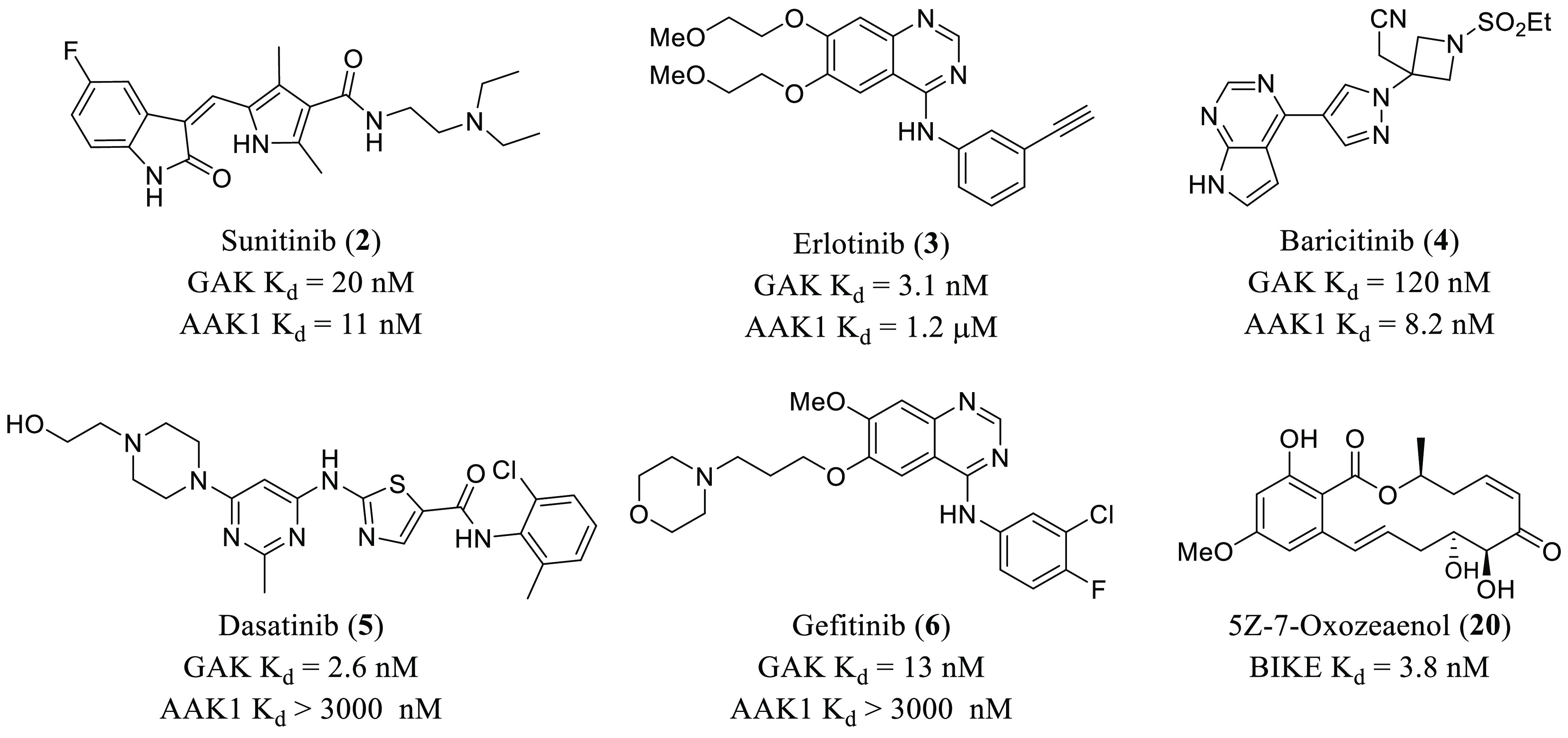
Chemical structures of GAK, AAK1, and BIKE inhibitors with antiviral activity.

GAK and AAK1 inhibitors, such as sunitinib (**2**) and erlotinib (**3**) (Figure 2 and Table 1),^53^ that were previously developed for other indications have been reported to have broad-spectrum antiviral activity against distant RNA viruses belonging to the *Flaviviridae*, *Filoviridae*, *Togaviridae*, *Arenaviridae*, or *Paramyxoviridae* families.^49,52^ Moreover, baricitinib (**4**, Figure 2 and Table 1),^42^ a potent GAK and AAK1 inhibitor, has been proposed as an effective therapy for coronavirus disease 2019 (COVID-19).^54^ In fact, treatment of COVID-19 pneumonia patients with **4** decreased ICU hospitalization and improved clinical parameters.^55^ However, its ability to also inhibit janus kinase (JNK1/2) could indicate other risks that need to be considered,^56^ despite being favorable for managing inflammation. Thus, only short treatments (7–14 days) with **4** are recommended to avoid opportunistic viral infections.^57^ To our knowledge, the first known inhibitors of AAK1 and GAK, rather than being selective, were multikinase inhibitors initially developed to inhibit other kinases. This is the case for sunitinib (**2**), erlotinib (**3**), baricitinib (**4**), dasatinib (**5**), or gefitinib (**6**), among others (Figure 2 and Table 1).

**Table 1:** Overview of Representative PKIs and LKIs and Their Effect on Virus Infection

inhibitor	target	virus	affected stage/effect
sunitinib (**2**)	GAK/AAK1	HCV	entry
			assembly
erlotinib (**3**)	GAK/AAK1	HCV	entry
			assembly
	EGFR	HCV	entry
baricitinib (**4**)	GAK/AAK1	SARS-CoV-2	entry
			assembly
dasatinib (**5**)	Src	DENV	assembly and secretion
	Src	HCV	drop of IC_50_ 210-fold (in Huh7.5.1 cells in combination with sofosbuvir)
	Fyn	DENV	replication
	Lck	HIV-1	entry
			reverse transcription
gefitinib (**6**)	EGFR	TGEV	entry
		IAV	infection
		rhinovirus	
selumetinib (**9**)	MEK1/2	MERS-CoV	inhibitory effects
trametinib (**10**)	MEK1/2	MERS-CoV	inhibitory effects
palbociclib (**14**)	CDK4/6	HIV-1	replication
		HSV-1	reverse transcription
apilimod (**18**)	PIKfyve	EBOV	entry
		SARS-CoV-2	entry
genistein (**36**)	various RTKs	IAV	entry
		HIV-1	replication
		arenavirus	
		HSV-1	
R428 (**38**)	Axl	ZIKV	entry
			activates IFN-1
AG879 (**39**)	TrKA	IAV	replication
tyrphostin A9 (**40**)	PDGFR	IAV	replication
		TGEV	replication
U0126 (**41**)	MEK1/2	IAV	vRNP export
		astrovirus	all stages
		MERS-CoV	entry
SP600125 (**42**)	JNK-1	JEV	reduction of inflammatory cytokines
SB203580 (**43**)	p38	MERS-CoV	entry
papaverine (**44**)		IAV	vRNP export
		paramyxovirus	
ATR-002 (**45**)	MEK1	IAV	antiviral activity
		IBV	
saracatinib (**46**)	Src	DENV	assembly
	Fyn	DENV	replication
(*R*)-roscovitine (**47**)	CDK2/5	HCMV	DNA synthesis
flavopiridol (**48**)	CDK1/2/4	HIV-1	replication
		IAV	antiviral activity (synergistic effect with dinaciclib, **51**)
alsterpaullone (**49**)	CDK1/2	HIV-1	cell viability
FIT-039 (**50**)	CDK9	HSV-1	replication
			transcription
		HSV-1	replication
		HCMV	
		HAdV-5	
PHA-690509 (**52**)	CDK2	ZIKV	replication
YM201636 (**53**)	PIKfyve	EBOV	entry
vacuolin-1 (**54**)	PIKfyve	EBOV	entry
GNF-2 (**57**)	ABL	DENV	entry
			replication
BSA9 (**58**)	CaMKII	DENV	entry
		ZIKV	
dorsomorphin (**60**)	AMPK	EBOV	replication

**Figure 3:**
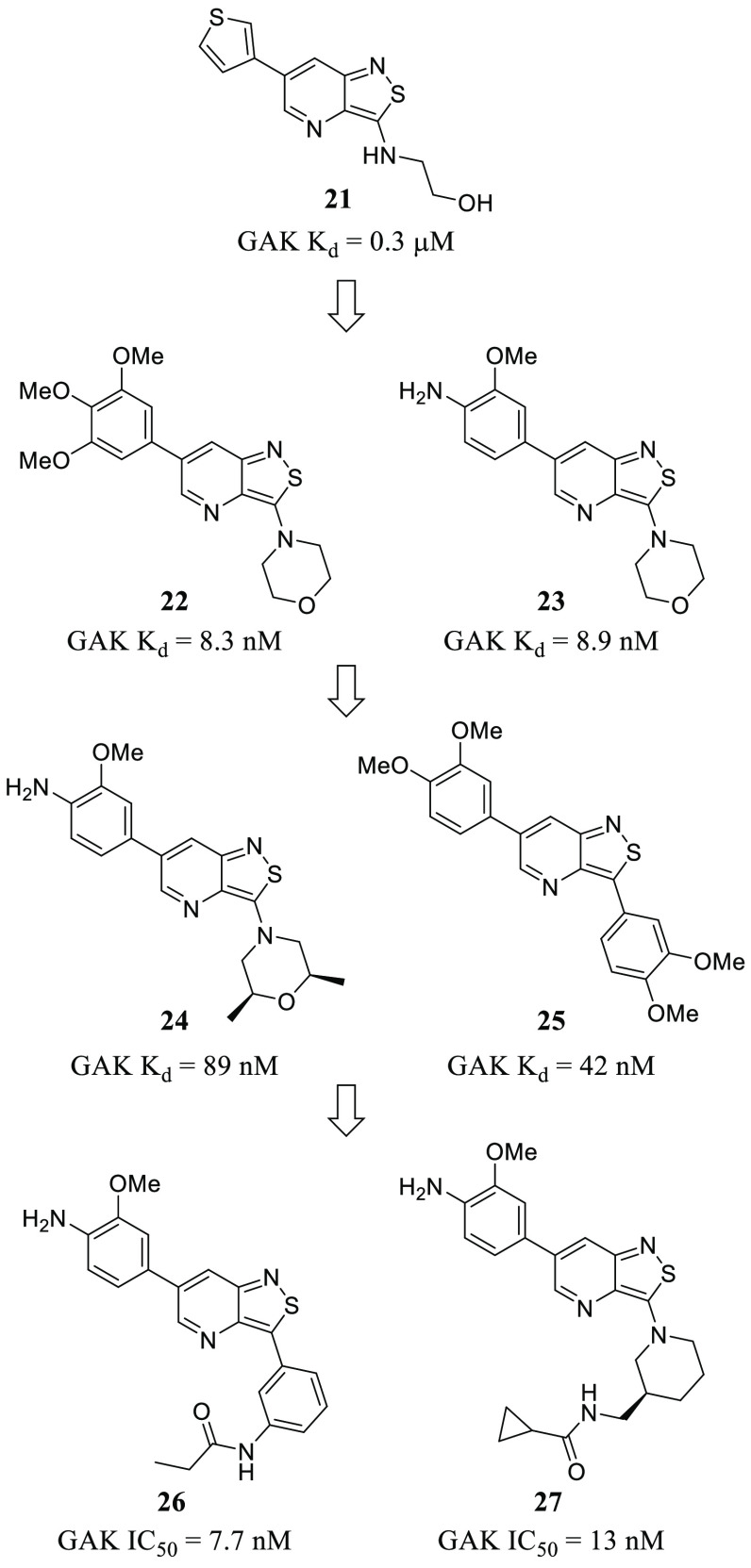
Chemical structures of 3,6-disubstituted isothiazolo[4,3-*b*]pyridines **21**–**27** with antiviral activity.

With respect to new chemical entities optimized and developed as antivirals, there have been remarkable efforts to improve the chemical space. These efforts have focused on the development of selective inhibitors. In this sense, the discovery of a hit bearing a isothiazolo[4,3-*b*]pyridine scaffold with *K*_d_ = 0.3 μM in GAK (**21**, Figure 3) has been reported.^58^ Structural variation at positions 3 and 6 led to optimized derivatives (**22**–**25**, Figure 3) with stronger affinities for GAK in the nanomolar range.^58−60^ It was seen that an aromatic moiety at position 6, particularly 3,4,5-trimethoxyphenyl and 2-methoxy-3-aminophenyl, while maintaining a morpholino group at position 3 is the optimal substitution for a potent and selective inhibition of GAK. Moreover, further exploration around position 3 revealed that methyl-substituted morpholin analogues were optimal for GAK inhibition together with an antiviral effect.^59^ The chemical diversity of this novel series of GAK inhibitors can be significantly improved with the newly reported possibilities that carbon-linked substituents at position 3^60^ and even modifications in the pyridine moiety^61^ offer. The most potent compound of this new series was derivative **25** (Figure 3) that bears a 3-(3,4-dimethoxyphenyl) group at position 3 and presents a strong affinity for GAK (*K*_d_ = 42 nM) together with moderate activity against DENV (EC_50_ = 3.4 μM).^60^ Reported molecular docking studies showed that the 3,4-dimethoxyphenyl group at position 3 forms a hydrogen bond with Lys69 at the ATP binding site, while the 3,4-dimethoxyphenyl group at position 6 is oriented to establish a hydrogen bond with Arg44. These interactions offer new possibilities for optimization. In fact, different phenyl-substituted and *N*-piperidinyl moieties were introduced at position 3 of the main isothiazolo[4,3-*b*]pyridine scaffold in order to increase GAK affinity by hydrogen bond interaction with Lys69. Optimized compounds (**26** and **27**, Figure 3) not only showed GAK inhibition in the nanomolar range (IC_50_ = 7.7 nM and 13 nM, respectively) but also anti-DENV properties in the sub-micromolar range (EC_50_ = 1.9 μM and 7.5 μM, respectively).^62^ Besides 3,6-disubstituted isothiazolo[4,3-*b*]pyridines, other new scaffolds developed as selective GAK inhibitors, particularly, 4-anilinoquinolines and 4-anilinoquinazolines, showing potential antiviral properties have been reported, although the structure–activity relationship (SAR) has not being so extensively studied.^63,64^

**Figure 4:**
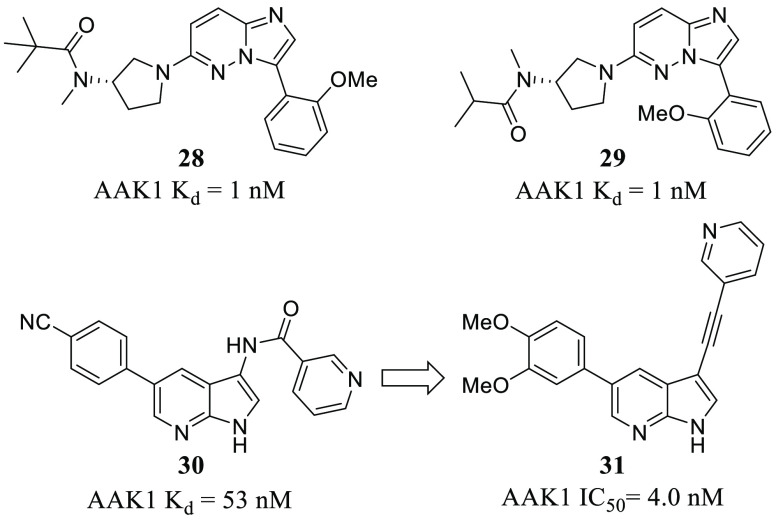
Chemical structures of imidazo[1,2-*b*]pyridazines **28** and **29**, and pyrrolo[2,3-*b*]pyridines **30** and **31** with antiviral activity.

Regarding AAK1, as this enzyme was considered as a pharmacological target for neurological disorders, different chemotypes were developed as drugs for these diseases. Such is the case for a series of imidazo[1,2-*b*]pyridazines. Although these were originally described for neuropathic pain therapy,^65^ some derivatives showed antiviral activity against HCV and dengue virus (DENV) (**28** and **29**, Figure 4).^52^ On the basis of this proof-of-concept, a potent AAK1 inhibitor (**30**, *K*_d_ = 53 nM, Figure 4) bearing a pyrrolo[2,3-*b*]pyridine scaffold was identified after a screening campaign using 577 chemically diverse kinase inhibitors and 200 protein kinases.^66^ It was chosen for optimization considering also antiviral properties. Chemical variations around this main core led to new derivatives in the low nanomolar range with AAK1 inhibition and improved activity against DENV (**31**, Figure 4). EC_50_ for DENV was 8 μM (compound **30**) vs 0.72 μM (compound **31**).^67^ With respect to kinase selectivity, although not highly selective, optimized candidates presented a better profile than sunitinib (**2**, Figure 2), and they are useful pharmacological tools to be used instead of this drug.

Besides the above-mentioned role of AAK1 and GAK in antiviral infection, another member of the NAK family, BIKE, has been validated as a druggable target for the development of broad-spectrum antivirals. The proof-of-concept of this assertion is based on the suppression of DENV infection by pharmacological inhibition of BIKE using a known nonselective inhibitor, the natural product 5*Z*-7-oxozeaenol (**20**, Figure 2), that was developed as an antitumoral drug. BIKE is required for both early and late stages of the DENV life cycle. Importantly, this effect is partly mediated by the phosphorylation of the Thr156 residue of AP2M1.^68^

**Figure 5:**
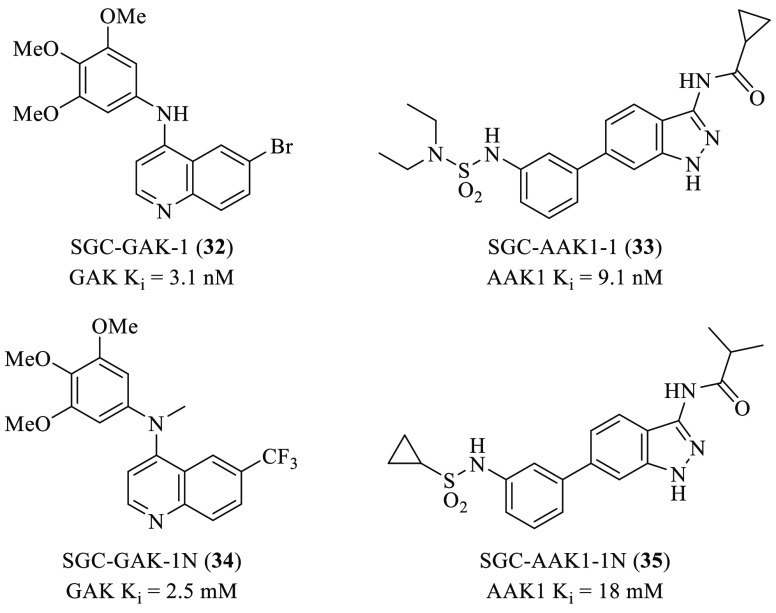
Chemical structures for GAK and AAK1 probes **32** and **33**, and their structurally related negative controls **34** and **35**, respectively.

Further studies focused on the development of selective NAK inhibitors are needed to improve the knowledge around the NAK family members and to confirm their potential as broad-spectrum antivirals. In this regard, the use of chemical probes such SGC-GAK1^69^ (**32**, Figure 5) or SGC-AAK-1^70,71^ (**33**, Figure 5) will be of utmost importance. It is also noteworthy that the structurally related negative controls, **34** and 35, are available (Figure 5).^69,71^

## 3 Receptor Tyrosine Kinases (RTKs)

Receptor tyrosine kinases (RTKs) are cell surface receptors that regulate key cellular processes, such as cell cycle control, metabolism, and cell differentiation, through downstream signaling pathways. There are 58 human RTKs that are classified into 20 subfamilies. All of them have a similar architecture: an extracellular region (usually glycosylated) for ligand binding, a transmembrane helix, and an intracellular part bearing the protein tyrosine kinase domain. Additionally, the mechanism of activation is highly conserved. It is based on a ligand-induced dimerization/oligomerization that produces the activation of downstream signaling pathways inside the cell. Because of the key biological role of this family of kinases, mutations and deregulations have been linked to diseases such as cancers, diabetes, inflammation, severe bone disorders, arteriosclerosis, and angiogenesis.^72−75^

**Figure 6:**
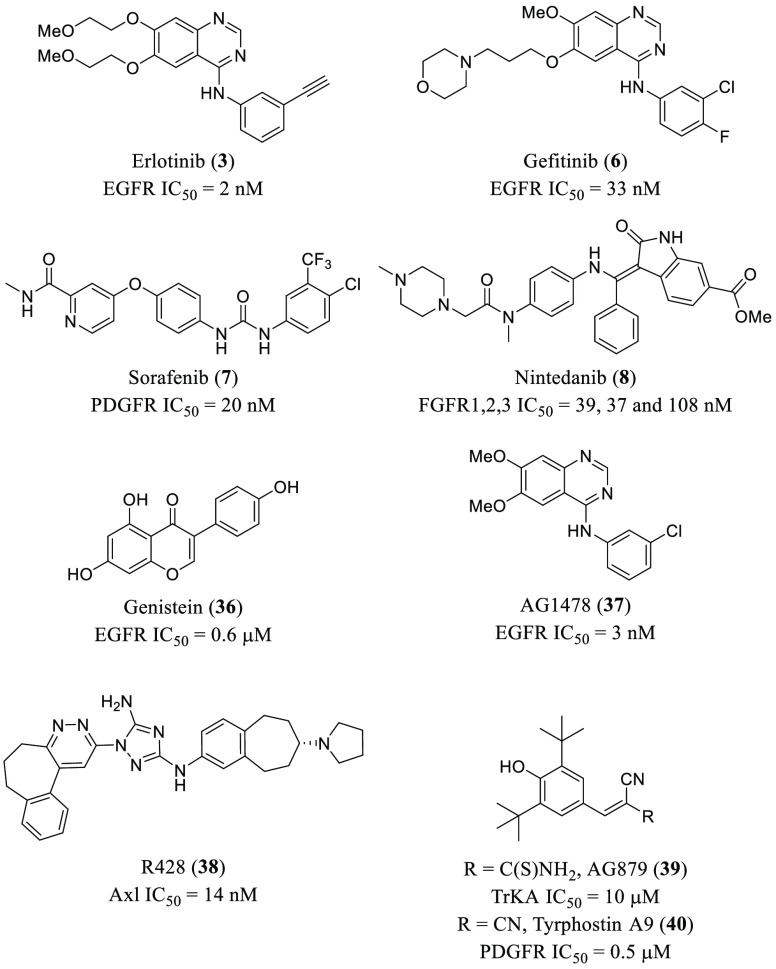
Chemical structures of representative RTK inhibitors with antiviral activity.

RTKs have also been proven to be important factors implicated in the infection process of some viruses. In this sense, the epidermal growth factor receptor (EGFR) is a member of the ErbB subfamily of RTKs, and it has been the most studied.^76^ There are reports supporting that its activation is necessary for the entry of various viruses. For example, binding of cell culture HCV particles (HCVcc) to both primary human hepatocyte (PHH) cells or antibody-mediated cross-linking of CD81 induces EGFR activation, which is required for HCV clathrin-mediated endocytosis.^77,78^ While incubation of Huh-7.5 cells with EGFR ligands epidermal growth factor (EGF) and transforming growth factor-α (TGF-α) showed a higher infection rate by inducing EGFR internalization and colocalization with CD81, it did not have any effect on HCVcc infection if ligands were added after the viral genome had entered cells. Additionally, EGFR inhibitor erlotinib (**3**, Figure 6 and Table 1) prevented HCVcc infection of Huh-7.5 cells. These results altogether suggest that endocytosis of EGFR is essential for HCV entry, although it does not have any effect on HCV RNA replication. It should be noted that the actual molecular mechanisms of EGFR-mediated endocytosis still remain unclear. Both genistein (**36**, Figure 6 and Table 1), a general inhibitor of RTKs, and EGFR narrow spectrum inhibitor gefitinib (**6**, Figure 6 and Table 1), impair influenza A virus (IAV) entry in A549 lung epithelial cells.^79^ The selective EGFR inhibitor AG1478 (**37**, Figure 6) has been shown to block transmissible gastroenteritis virus (TGEV) uptake by porcine intestinal columnar epithelial cells (IPEC), and it has a negative impact in IAV and rhinovirus infection of epithelial BEAS-2b cells.^80^ Recently, EGFR inhibitors such as erlotinib (**3**) or gefitinib (**6**) (Figure 6) and platelet-derived growth factor receptor (PDGFR) inhibitor sorafenib (**7**, Figures 1 and 6) have been proposed to prevent the excessive fibrotic response in different human coronavirus infections, including severe acute respiratory syndrome CoV-2 (SARS-CoV-2), but the beneficial effect of these kinase inhibitors is not associated directly with any antiviral activity.^81^ In fact, nintedanib (**8**, Figures 1 and 6), a fibroblast growth factor receptor (FGFR) inhibitor, is in clinical trials to treat pulmonary fibrosis in mild COVID-19 patients (NCT04338802).

Axl belongs to the Tyro3 Axl Mer (TAM) RTK family, and it is implicated in the clearance of apoptotic cells and regulation of innate immunity.^82^ ZIKA virus (ZIKV) enters via clathrin-mediated endocytosis in human glial cells expressing the Axl receptor. Blocking its kinase activity by the small-molecule inhibitor R428 (**38**, Figure 6 and Table 1) avoids viral entry and increases type 1 interferon (IFN-1) signaling.^83^ Similarly, the use of anti-Axl antibodies (Ab) completely inhibited the entry of the dengue virus (DENV) in different cell lines. A549, Vero, Cos-7, and Huh7 5.1 cells showed high levels of Axl receptor and infection by DENV occurred rapidly. In contrast, 293T, U937, or RAJI cells, all of which lack the receptor, were poorly susceptible to infection.^84^ Axl mediates DENV infection not only by enhancing virus endocytosis but also by initiating downstream signaling pathways that facilitate infection. Additionally, Axl has recently been reported to mediate endocytosis of SARS-CoV-2, which causes the current COVID-19 pandemic.^85^ Results show that blocking the Axl receptor decreases viral infection in H1299 pulmonary and human primary lung epithelial cells, and protein levels in COVID-19 patients correlate with the levels of spike protein of SARS-CoV-2 in bronchoalveolar fluid cells.

In addition to preventing the entry of the virus into the cells, RTK inhibitors are also capable of affecting other stages of the viral life cycle. For instance, genistein (**36**, Figure 6 and Table 1) has been shown to block replication of human immunodeficiency virus-1 (HIV-1),^86^ arenavirus,^87^ and herpes simplex virus type 1 (HSV-1).^88^ The replication of IAV has also been disrupted by the inhibitors AG879 (tropomyosin receptor kinase A and human epidermal growth factor receptor 2-TrKA/HER2-inhibitor, **39**, Figure 6 and Table 1) and tyrphostin A9 (PDGFR, **40**, Figure 6 and Table 1), even though the mechanism of action and the specific targets are still unclear.^89^ A high-throughput screening (HTS) campaign of approximately 1300 compounds also found that tyrphostin A9 (**40**, Figure 6 and Table 1) is able to block replication of TGEV in PK-15 or ST cells.^90^

## 4 Mitogen-Activated Protein Kinases (MAPKs)

**Figure 7:**
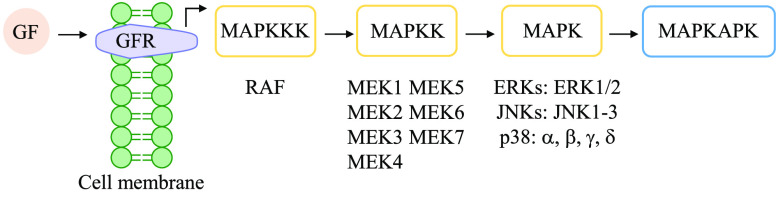
MAPK signaling cascade upon binding of a GF to its receptor, leading to the activation of MAPKAPK.

The mitogen-activated protein kinase (MAPK) signaling pathway encompasses a complex network of phosphorylation cascades which transduce external stimuli into a wide range of cellular responses. Generally, the cascade is initiated by the interaction of a growth factor (GF) with its specific receptor (GFR), which drives the phosphorylation of MAPK kinase kinases (MAPKKKs) by a protein of the Ras/Rho family. The activation of MAPKKKs, which are Ser/Thr kinases, subsequently phosphorylates and activates MAPK kinases (MAPKKs, with seven MEK isoforms) and finally stimulates MAPK activity through dual phosphorylation on Thr and Tyr residues within a conserved Thr-X-Tyr motif located in the activation loop of the kinase domain (Figure 7). There are three important families within the MAPKs: ERKs (with ERK1 and ERK2 isoforms), JNKs (JNK1, JNK2, and JNK3 isoforms), and p38 MAPKs (with p38α, p38β, p38γ, and p38δ isoforms). Activated MAPKs lead to the activation of downstream proteins (MAPK-activated protein kinases, MAPKAPK) that produce the amplification of key molecules (mostly transcription factors) that are involved in important biological processes such as mitosis, metabolism, motility, survival, apoptosis, and differentiation (Figure 7).^91,92^

**Figure 8:**
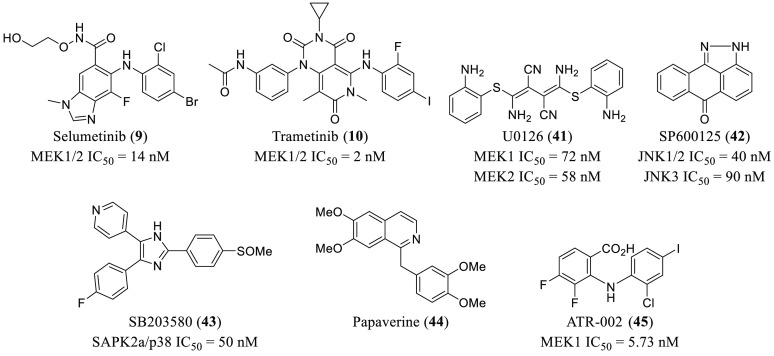
Chemical structures of representative MAPK inhibitors with antiviral activities.

In the late 90s, some DNA and RNA viruses were proven to induce the MAPK cascade in infected cells.^93,94^ However, it was not until the turn of the millennium when Stephan Ludwig and co-workers disclosed an important breakthrough. They showed that proliferation of IAV was impaired by the inhibition of the RAF/MEK/ERK signaling pathway, which seems to be essential for virus production and ribonucleoprotein (RNP) export from the nucleus during the viral life cycle.^95^ They were able to show that treatment of infected Madin-Darby canine kidney (MDCK) cells with MEK1/2 inhibitor U0126 (**41**, Figure 8 and Table 1) at 50 μM produced a decrease of virus titers up to 80%. This effect was observed at a multiplicity of infection (MOI) values of 0.0025 and 1.0 after 48 and 9 h, respectively. A reduction in the number of virus particles was also observed when a dominant negative mutant of ERK (ERK(2C3)) was transiently expressed. Importantly, whereas the synthesis of viral RNA or proteins was not affected by **41** treatments, viral RNP (vRNP) complexes were unable to exit the nucleus, inhibiting virus production. Later, they were also able to demonstrate that proliferation of both IAV and influenza B virus (IBV) was inhibited in MDCK cells by the treatment of **41** without the emergence of resistant variants,^96^ including the H1N1 pandemic strain.^97^ This MEK1/2 inhibitor was additionally useful for the suppression of astrovirus replication in Caco-2 cells, and it was effective at all the stages of the viral life cycle.^98^

As discussed in the Introduction, quantitative phosphoproteomic analysis is a recent methodology aimed at the study of phosphorylation profiles. In 2016, a study reported the use of this strategy to identify SP600125 (**42**, Figure 8 and Table 1), a JNK-1 inhibitor, as a potential therapeutic solution for the infection of Japanese encephalitis virus (JEV).^40^ They examined the phosphorylation pattern in U251 cells upon JEV infection, and a number of the 604 proteins were differentially regulated compared to mock-infected cells. Then, the use of bioinformatic tools, such as the Ingenuity Pathway Analysis, allowed identification of JNKs as a protein kinase with a critical role in the infection. Experimental work to gain more insight into relationship between JEV infection and JNK-1 activation showed that JEV RNA genome extracted from infected cells participated in JNK-1 phosphorylation in a dose-dependent manner. Pharmacological inhibition of JNK-1 with derivative **42** produced a significant reduction of the inflammatory cytokines produced by JEV-infected U251 and BV-2 cells. Notably, they were able to reproduce the antiviral effect of **42***in vivo*. Quantification of the viral load in the brains of mice infected with JEV not only revealed a relevant decrease of infection but also an increased survival rate.

Kinome (the complete set of protein kinases encoded in its genome) analysis has also proved to be useful in the identification of host signaling networks that are important for Middle East respiratory syndrome coronavirus (MERS-CoV) infection.^99^ In this study, they performed a temporal kinome analysis in Huh7 human hepatocytes infected with MERS-CoV. They found that the MAPK pathway (among others) has a pivotal role in the early stages of the infection. Briefly, incubation of a certain kinome (cell lysates) with immobilized peptide arrays as targets produces the phosphorylation of some of the substrates. Once the phosphorylated peptides are visualized using either radioactive ATP or specific phosphoprotein stains, the active kinases can be determined.^100^ They disclosed that both SB203580 (p38 inhibitor, **43**, Figure 8 and Table 1) and U0126 (MEK1/2 inhibitor, **41**, Figure 8) are capable of preventing infection by 45% and 51%, respectively, at 10 μM. In addition, U0126 (**41**) produced a much higher antiviral activity in preinfected cells when compared to its addition after 2 h of the infection. Importantly, the licensed drugs selumetinib (**9**) and trametinib (**10**) (Figures 1 and 8 and Table 1), which target the MAPK pathway, had outstanding inhibitory effects (>95%), making drug repurposing of existing medicines a useful strategy toward the treatment of MERS-CoV infection.

Very recently, a drug repurposing strategy allowed the identification of the alkaloid papaverine (**44**, Figure 8 and Table 1) as a therapeutic agent with antiviral properties against influenza viruses and paramyxoviruses.^101^ They selected various natural products (20) present in plants and known to have antiviral properties and evaluated them in infected HEK293T cells at 50 μM. Among them, **44** showed the best activity against different influenza virus and paramyxovirus strains, with IC_50_ values ranging from 2.0 to 36.4 μM. Upon treatment of infected cells with **44**, the phosphorylation of MEK and ERK was reduced, while the total amount of protein remained unchanged. Additionally, inhibition of the MEK/ERK signaling pathway prevents vRNPs export from the nucleus, whereas it does not suppress viral RNA synthesis. These data suggest that the natural product papaverine exerts its effect at the end of the viral life cycle.

Atriva Therapeutics has discovered ATR-002 (**45**, Figure 8 and Table 1), an MEK inhibitor with antiviral properties. Compound **45** is the active metabolite of CI-1040, a compound that was previously used in clinical studies against cancer but was abandoned due to low plasma concentration presence. At that stage, the development of **45** was also discontinued, even though it had a lower IC_50_ than CI-1040 for MEK inhibition (5.73 vs 17.00 nM).^102^ However, **45** has been reported to have broad antiviral activity against different influenza virus strains of IAV and IBV, both in cell culture and in a mouse model.^103^ It has already successfully passed a Phase I clinical trial in 70 healthy subjects (Clinical trial identifier: NCT04385420).^104^ Notably, Atriva Therapeutics is currently carrying out a double-blind, randomized Phase II trial designed to evaluate **45** as a treatment for COVID-19 (EU Clinical Trial Register Number: 2020-004206-59), the disease caused by SARS-CoV-2.

## 5 Src Kinases

**Figure 9:**
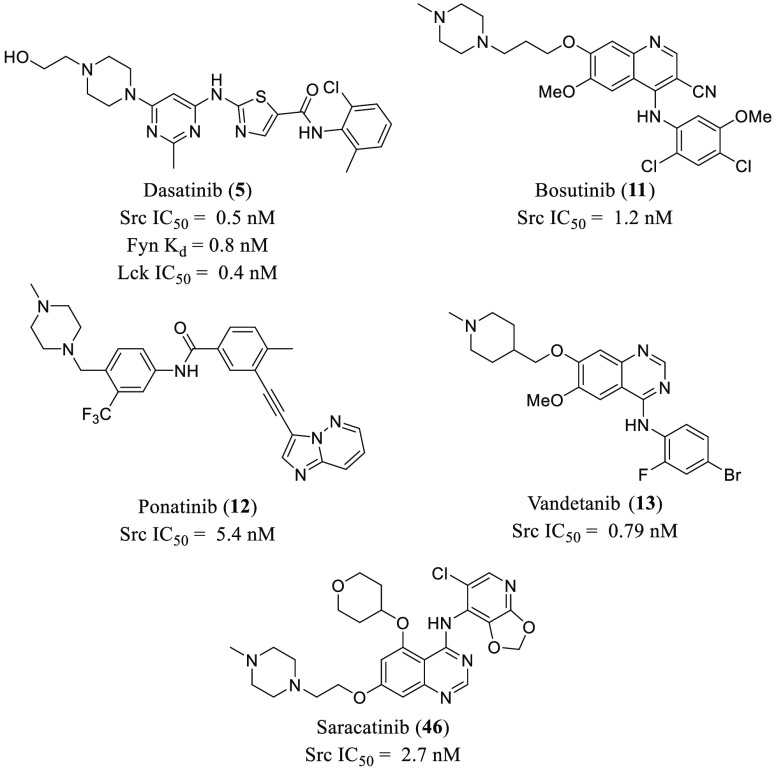
Chemical structures of FDA-approved Src kinase inhibitors with antiviral activity.

Src’s are nonreceptor Tyr kinases that are implicated in important cell physiological processes such as motility, differentiation, cell cycle progression, and survival, among others. There are 11 members in this family, classified in three different groups (I: Src, Fyn, Yes, and Fgr; II: Blk, Hck, Lck, and Lyn; III: Frk, Srm, and Brk).^4^ Src inhibitors have been mainly studied in the oncology field. In fact, there are four FDA-approved compounds aimed at the treatment of different types of cancer: dasatinib (**5**), bosutinib (**11**), ponatinib (**12**), and vandetanib (**13**) (Figure 1). Nevertheless, Src kinases are not the main players in the development of tumor malignancies, so a multidrug therapy is needed.^105^ Targeting Src protein kinases has also been investigated for the treatment of other pathologies, such as for immunotherapy or viral infections.^106^ For instance, in 2019 saracatinib (**46**, Figure 9) was granted the Orphan Drug Designation by the FDA for the treatment of idiopathic pulmonary fibrosis and an early Phase I clinical trial for the treatment Parkinson’s disease psychosis is currently recruiting volunteers (Clinical trial identifier: NCT03661125).^104^ Saracatinib (**46**, Figure 9 and Table 1) inhibits MERS-CoV at early stages of the viral cycle with an estimated EC_50_ of 2.9 μM and a CC_50_ of >50 μM and other human coronaviruses such as hCoV-229E and OC43 with an EC_50_ of 2.4 μM and 5.1 μM, respectively.^107^

Regarding Src inhibitors targeting viral infections, dasatinib (**5**, Figure 9 and Table 1) has been the main player in this field so far. For instance, an immunofluorescence screening assay of 120 mammalian protein kinases showed a strong relationship between Src inhibition and anti-DENV activity.^108^ Among the PKIs evaluated, **5** and **46** (Figure 9 and Table 1) displayed the best inhibition profile. They reduced DENV infection in Vero, Huh-7, and C6/36 cells in a dose-dependent manner from 0.05 to 5 μM. Notably, silencing the expression of Src protein using a small interfering RNA (siRNA) produced a significant drop in DENV titers 3 days after the infection, suggesting a key role of the Src kinase family in the DENV infection process. Specifically, Src kinase is necessary for the assembly and secretion of the virus, since immunofluorescence staining showed labeled DENV envelope protein accumulations within the perinuclear region but not throughout the cytoplasm and plasma membrane of the dasatinib-treated DENV-infected cells. Another recent study with **5** and **46** reported inhibition of DENV RNA replication via Fyn kinase.^109^

A lot of efforts have been made toward the identification of Src inhibitors for the treatment of HIV-1 infection. Lck kinase promotes T-cell lymphocyte activation through the activation of various transcription factors, such as NF-κB, NFAT and AP-1, which are essential for HIV-1 replication.^110^ In fact, an immunomodulation strategy using dasatinib (**5**, Figure 9 and Table 1) has been proven to control HIV-1 viral replication through downregulation of T-cell proliferation by using antigens derived from trivalent influenza vaccine.^111^ Regarding its mechanism of action, there are reports supporting that **5** blocks HIV-1 infection both during the entry at the hemifusion step and downstream by targeting the cellular restriction factor SAMHD1 for dephosphorylation. In the first case, treatment of U87.CD4.CCR5 cells with 300 nM of **5** decreased cell–cell fusion with HIV-1-envelope-expressing cells by 93%.^112^ Nonetheless, **5** was ineffective in inhibiting HIV-1 fusion with peripheral blood lymphocytes (PBMC) at any concentration assayed, but it was able to completely impair HIV-1 reverse transcription in the same cell line.^113^ Even though blocking Src kinases may have a great advantage in the treatment of HIV-1 over immunosuppression therapies, the risk for increased toxicity has to be carefully evaluated.^110^

Interestingly, a multidrug strategy has been carried out which found that the use of a combination of Src kinase inhibitor **5** with the viral entry inhibitor sofosbuvir showed synergistic effects in the treatment of HCV. This potent combination produced a drop in sofosbuvir’s IC_50_ up to 210-fold in Huh7.5.1 cells, thus improving the antiviral activity against HCV.^114^

## 6 Cyclin-Dependent Kinases

Cyclin-dependent kinases (CDKs) phosphorylate Ser/Thr residues in substrate proteins upon binding with a cyclin regulatory protein whose levels are controlled during the cell cycle. When a cyclin binds to a CDK, there is a conformational change that allows additional interactions of the kinase with the ATP in the catalytic domain so that phosphorylation can take place. The most important CDKs can be grouped according to their biological role. They are mainly involved in cell cycle regulation and gene transcription. There are three subfamilies related to the regulation of the cell cycle (CDK1, CDK4, and CDK5) that can bind multiple cyclins, and there are five more conserved transcriptional subfamilies (CDK7, CDK8, CDK9, CDK11, and CDK20) activated by a single cyclin.^115^ Regarding their specificity, CDK inhibitors can be classified as nonspecific (not pharmacologically relevant), pan-specific (they preferentially inhibit CDKs, but they are not very selective), oligospecific (they only inhibit a subset of CDKs), and monospecific (specific against one CDK). Additionally, they can be grouped considering their chemical structure as purine-type and non-purine-type inhibitors.^116^ Because CDKs are usually redundant regarding their biological functions, inhibition of a single CDK may be insufficient. Thus, searching for pan- or oligospecific inhibitors is usually a more fruitful approach than aiming for monospecific blockers.^117^ Traditionally, blocking CDKs has been a popular strategy for the treatment of many types of cancer.^118^ In fact, palbociclib (**14**), ribociclib (**15**), and abemaciclib (**16**) (Figure 1) are the only FDA-approved CDK inhibitors that are prescribed as breast cancer medications.^119^ Compound **14** inhibits HIV-1 and HSV-1 replication *in vitro* with EC_50_ values of 0.016 μM and 0.020 μM, respectively. This is partially due to its CDK6 inhibitory properties (IC_50_ = 0.016 μM).^120^ Recently, compound **16** has shown *in vitro* activity against SARS-CoV-2 (IC_50_ = 6.6 mM, CC_50_ > 50 mM).^121^

**Figure 10:**
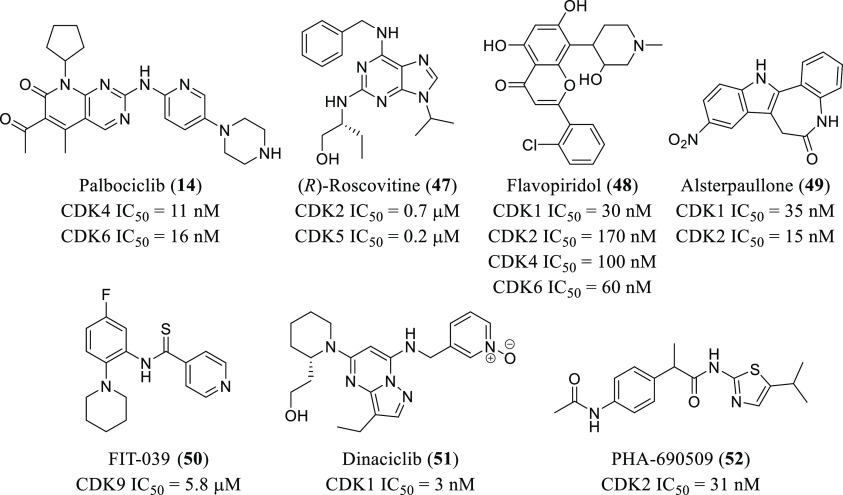
Chemical structures of the first molecules identified as CDK inhibitors with antiviral activities.

The purine analogue CDK inhibitor (*R*)-roscovitine (**47**, Figure 10 and Table 1) was first reported to impair human cytomegalovirus (HCMV) DNA synthesis in 1997.^122^ Since then, other CDK inhibitors (both purine- and non-purine-type) have been proven to have antiviral activities against a wide range of viruses.^123^ These agents exert their therapeutic action mainly by impairing viral transcription in infected cells. The selectivity against viral versus cellular transcription is not yet fully characterized and understood.

The inhibition of CDKs has been extensively studied for the treatment of HIV-1 infection. In the early 2000s, the CDK1/2/4 inhibitor flavopiridol (**48**, Figure 10 and Table 1) was one of the first CDK inhibitors disclosed to affect HIV-1 replication by impairing Tat-activated transcription.^124^ In 2010, Kashanchi and co-workers reported a small screening of CDK inhibitors for the identification of agents with antiviral activity against HIV-1.^125^ A set of 24 molecules was assayed in various cell lines by measuring cell viability after 74 h of the HIV-1 infection. The purine analogue alsterpaullone (**49**, Figure 10 and Table 1) was identified as the best compound. It showed a dose-dependent inhibition of viability in infected ACH2, OM10.1, J1-1, and U1 cells and good selectivity compared to the control uninfected group. By using [γ-^32^P]-labeled histone H1 as substrate for autoradiography visualization, immunoprecipitation experiments revealed that **49** completely inhibited CDK2 kinase activity in infected cells at 0.5 μM,. The expression and protein levels of functional CDK2 were also downregulated using the inhibitor in HIV-1 infected cells. Importantly, a synergistic effect was observed when both **47** and **49** were used in PBMC infected cells, reinforcing the idea indicated previously that inhibition of multiple CDKs may be needed to achieve the desired effect. A recent study also showed that palbociclib (**14**, Figure 10 and Table 1), a potent CDK4/6 inhibitor, reduced phosphorylation of sterile α motif and HD domain-containing protein-1 (SAMHD1) in primary macrophages and CD4^+^ T lymphocytes, blocking HIV-1 reverse transcription and replication.^126^ This effect of **14** on SAMHD1 has also been observed in the case of HSV-1.^120^

Infections caused by DNA viruses have also been targeted using CDK inhibitors. For instance, an in-house kinase-directed library of 144 compounds was screened to identify selective CDK9 inhibitors as antiviral agents. As a result, compound FIT-039 (**50**, Figure 10 and Table 1) showed the best results with an IC_50_ of 5.8 μM, and it proved to be an ATP competitive inhibitor.^127^ In order to examine its antiviral properties, Vero cells were infected with HSV-1 at an MOI of 100, and viral replication was inhibited in a dose-dependent manner with no adverse effects on cell viability (EC_50_ and EC_80_ were 0.69 μM and 4.0 μM, respectively).

Additionally, **50** decreased phosphorylation of the carboxyterminal domain of RNA polymerase II in HSV-1 infected HEK293 cells, suggesting that the antiviral activity may be attributed to hampered viral transcription. Notably, when treated with FIT-039, mice presenting an HSV-1 infected skin lesion showed regression of the injury and survival, demonstrating *in vivo* effects. Compound **50** inhibited the replication of other DNA viruses, such as HSV-2, HCMV, human adenovirus (HAdV) type 5, and hepatitis B virus (HBV).^128^ Although the antiviral effect of **50** is acknowledged, the described biochemical IC_50_ value for the inhibition of CDK9 is somewhat higher than cellular EC_50_ and EC_80_ values, compromising the idea that the kinase inhibition is the unique mechanism of action.

HTS campaigns have also proved to be rewarding in discovering CDK inhibitors as therapeutic agents for the treatment of other viruses such as IAV or ZIKV. In the first case, flavopiridol (**48**, Figure 10 and Table 1) was identified from a 273 kinase inhibitor library, showing antiviral activity against various IAV strains in A549 cells with no cytotoxic effects.^37^ Even though they do not discuss the mechanism of action, they show an interesting synergistic effect using a combination of **48** (CDK1/2/4/6 inhibitor) and dinaciclib (CDK1/2/5/9 inhibitor, **51**, Figure 10). Interestingly, compound **51** has recently been identified as an antiviral agent in a quantitative mass spectrometry-based phosphoproteomics survey aimed at a study of the phosphorylation pattern produced by SARS-CoV-2 infection.^129^ Regarding the ZIKV, they use both the Library of Pharmacologically Active Compounds and the NCATS pharmaceutical collection, with a total of 4096 compounds.^130^ ZIKV infection in SNB-19 cells was inhibited by compound PHA-690509 (**52**, Figure 10 and Table 1) in a dose-dependent manner with an IC_50_ of 1.72 μM. In this report, they investigated the underlying mechanism by carrying out time-of-addition experiments which revealed that the reduction of viral RNA was only apparent after the entry phase and regardless the moment of addition of **52** (1 h before or 4 h after inoculation of the virus). The results all together led them to suggest that the identified hit compound produced the antiviral effect in the RNA replication step.

## 7 Phosphatidylinositol-3-Phosphate-5 Kinase (PIKfyve)

Phosphatidylinositol-3-phosphate-5 kinase (PIKfyve) is a lipid kinase located in the endosome membrane on the cytosolic face. It is responsible for the synthesis of phosphatidylinositol-3,5-biphosphate (PtdIns(3,5)P_2_) by phosphorylation of phosphatidylinositol-3-phosphate (PtdIns3P).^131^

Phosphoinositides are eukaryotic membrane phospholipids.^132^ They are markers for defining the identity of the endolysosomal subcompartments since they are restricted to specific intracellular membranes. The phosphorylated derivatives of phosphatidylinositol (PtdIns) previously mentioned are necessary for the endosome membrane dynamics and protein sorting.^133^ PtdIns3P is found in early endosome membranes, and it is involved in endosome maturation and multivesicular body biogenesis. In addition, PtdIns(3,5)P_2_ is a phosphoinositide implicated in various trafficking events associated with the endocytic pathway, it regulates the endosome maturation and it is involved in vacuole formation and size control. Although PtdIns(3,5)P_2_ represents only 1% of the phosphoinositides, it is essential for regulating of endosomal membrane homeostasis and the endolysosomal trafficking system.^131,134^

PIKfyve is crucial for lysosome fission and fusion between lysosomes and autophagosomes. These processes require a balance between PtdIns3P and PtdIns(3,5)P_2_ on the lysosomal membrane that is established by PIKfyve. Therefore, PIKfyve plays an essential role in maintaining cell morphology by regulating late endocytic membrane homeostasis.^135^ Because of this function, PIKfyve inhibition affects the early endocytic pathway.^131^ PIKfyve ablation causes endosomal swelling and vacuolation of late endosomes, and these changes could be caused by the decrease of the membrane fission and concomitant interference in endosomal traffic.^136,137^ Thus, PIKfyve seems to play multiple roles in trafficking events. PIKfyve inhibitors have been developed as cancer therapeutics.^138^ Being a selective PIKfyve inhibitor, apilimod (**18**, Figure 1)^139^ is in clinical trials for treatment of B-cell non-Hodgkin lymphoma.^140^ In addition, PIKfyve has been identified as a therapeutic target in amyotrophic lateral sclerosis (ALS).^141^ Modulation of vesicle trafficking by PIKfyve inhibitors rescues neurodegeneration due to the C9ORF72 repeat expansion.^142^ The role of PIKfyve in endosomal maturation, and therefore in trafficking events, has also been exploited for the development of antimicrobials.^143^

As mentioned above, virus entry initiates when the virus attaches to the host cell. The viral entry ends when the viral content reaches the cytosol. The binding process can occur following the endocytic uptake and, in this case, can use different initial trafficking routes to reach the site on membrane fusion. Moreover, the precise endosomal compartment used for virus penetration differs among viruses, and the pH threshold for activation allows the distinction between early and late-penetrating viruses that penetrate through the membranes of early (pH 6.5 to 6.0) and late endosomes (pH 6.0 to 5.5), respectively.^144^

Inhibition of PIKfyve has therefore been reported to have a negative effect in the infection by diverse viruses such as African swine fever virus (ASFV),^145^ Ebola virus (EBOV),^146^ or even SARS-CoV-2.^147^

**Figure 11:**
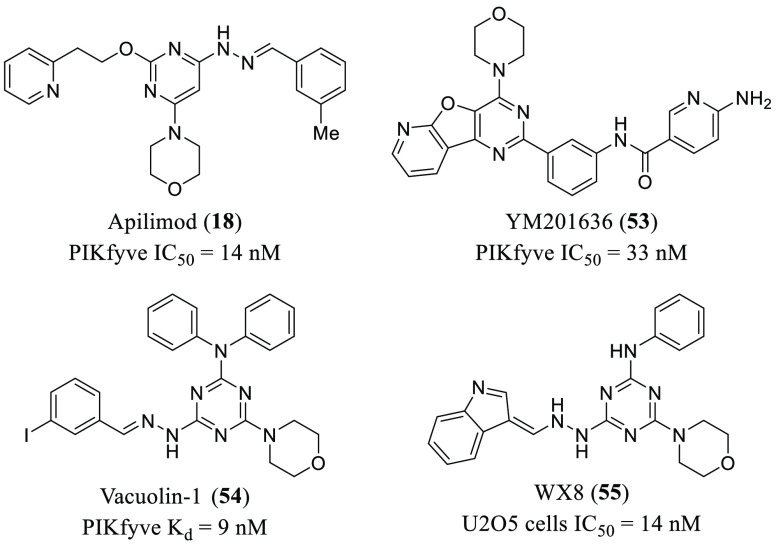
Chemical structures of representative PIKfyve inhibitors bearing a morpholino-azine core group.

EBOV requires engagement of the receptor protein Niemann-Pick C1 (NPC1) to enter into the host cell.^148^ This receptor is a polytopic protein that resides in the limiting membrane of late endosomes/lysosomes, which indicates the need for endosomal trafficking to reach the intracellular compartments containing NPC1.^146,147,149^ PIKfyve inhibition impeded colocalization of EBOV with NPC1 receptor and therefore entry into the cytoplasm. The requirement of PIKfyve for EBOV entry was demonstrated by using enzymatically dead mutants and also inhibitors of the enzyme such as apilimod (**18**), YM-201636 (**53**), and vacuolin-1 (**54**) (Figure 11 and Table 1). Remarkably, all pathogenic filoviruses were susceptible to PIKfyve inhibition.

Coronaviruses can enter into the cells by direct fusion at the plasma membrane or following receptor-mediated endocytosis, depending both on the protease TMPRSS2 and angiotensin converting enzyme 2 (ACE2) receptor.^150^ After binding of the SARS-CoV-2 spike (S) protein to the ACE2 receptor, the virus enters into the cell and is delivered to the endo-/lysosomal pathway.^151^ Thus, perturbation of the normal endosomal trafficking by PIKfyve inhibitors may block the entry of SARS-CoV-2.^147^ In fact, a phase 2 clinical trial is currently recruiting volunteers to study the effect of apilimod (**18**, Figure 11) in COVID-19 patients (NCT04446377).

The lack of PtdIns(3,5)P_2_ caused by PIKfyve inhibition is behind the antiviral effect of PIKfyve inhibitors. On one hand, this depletion produces endosomal swelling into small and spherical vacuoles, and, as consequence, the virus particles are retained in these vacuoles located adjacent to the endosomal limiting membrane and cannot fuse with it.^152^ On the other hand, virions might fuse with smaller intraluminal vesicles in the endosomal lumen, but the lack of PtdIns(3,5)P_2_ avoids the fusion with the endosomal limiting membrane and the subsequent release of virus genome into the cytosol.^153^

However, in spite of the antiviral effects exerted by PIKfyve inhibition, the number and chemical diversity of the reported inhibitors are few,^138^ most of them bear a morpholino-azine core group as the parent compound, apilimod^139^ (**18**, **53**–**55**, Figure 11).

The physiological effects described for these inhibitors include the inhibition of autophagy, reduced generation of IL-12/IL-23 and reduced dendritic cell infiltration in psoriasis.^154−156^ As mentioned above, some PIKfyve inhibitors also prevent viral infection. However, despite their potential as broad-spectrum antivirals, no specific programs to optimize antiviral properties of PIKfyve inhibitors have been reported. Moreover, almost all of the known inhibitors shared the same fundamental structure, that is, an azine core together with a common morpholine ring (Figure 11).

Therefore, since perturbing the common endosomal trafficking using PIKfyve inhibitors is thought to be the mechanism that may block entry of multiple viruses, the development of specific medicinal chemistry programs to optimize both enzyme inhibition and antiviral efficacy may be a promising strategy to obtain broad-spectrum antivirals targeting viruses that requires the endosomal pathway for infection.

## 8 Miscellaneous

### G Protein-Coupled Receptor Kinases (GRKs)

**Figure 12:**
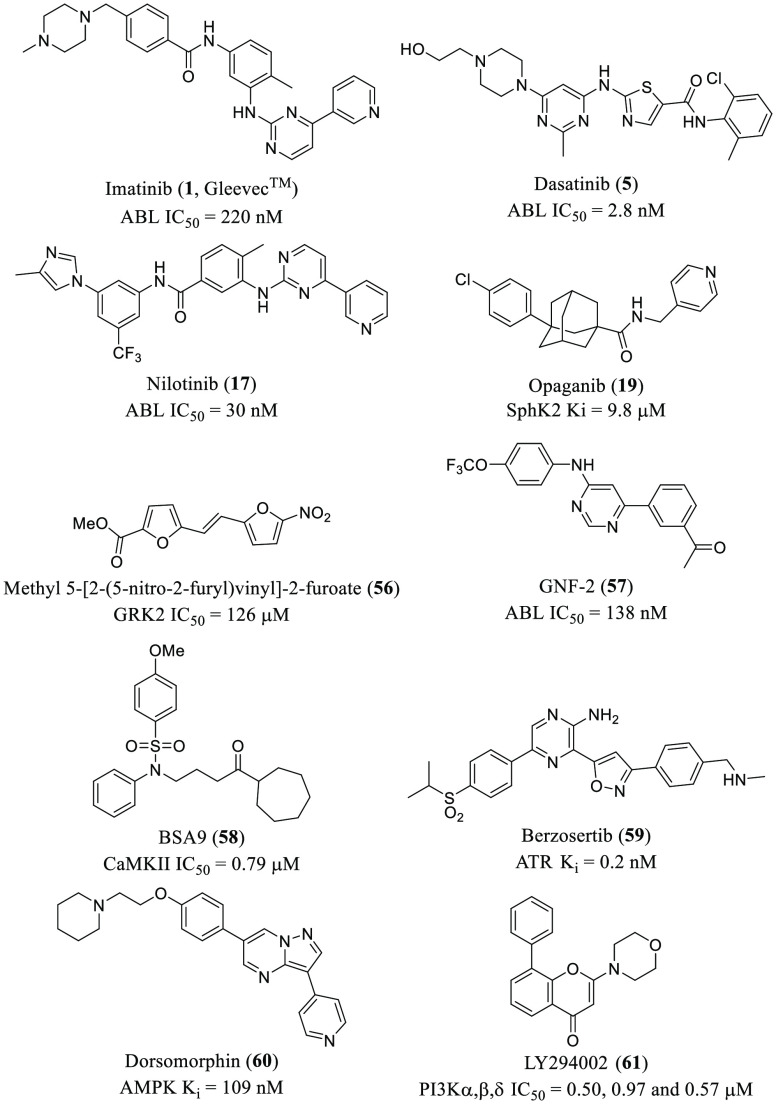
Chemical structures of inhibitors **1**, **5**, **17**, **19**, and **56**–**61** with antiviral activity.

GRKs modulate G protein-coupled receptor (GPCR) signaling through receptor phosphorylation, allowing the binding of arrestin proteins, which can lead to either receptor internalization or arrestin-mediated signaling cascades. GRKs are divided in three subfamilies based on their primary structure: visual family (GRK1 and GRK7), GRK2 family (GRK2 and GRK3), and GRK4 subfamily (GRK4, GRK5, and GRK6).^157^ GPCR signaling is involved in a wide range of biological functions, and its deregulation is associated with several pathologies. Thus, GRKs are popular therapeutic targets for the development of drugs to control GPCR signaling cascades. Regarding their antiviral activity, GRK2 inhibitors have recently been proposed as an efficient strategy to treat IAV infections. A phosphoproteomic-based kinase screening in A549 cells allowed the investigation of the phosphorylation changes that occurred in the kinome upon IAV infection.^41^ Quantification of the phosphorylation, which was carried out using stable isotope labeling with amino acids in cell culture (SILAC), revealed a unique phosphorylation signature induced by IAV entry (5–15 min postinfection). Among all the identified proteins, GRK2 was proposed to be a novel kinase activated in the early steps of the infection. Additional experiments validated that IAV infection induced activation of GRK2 and its translocation to the plasma membrane. Silencing the expression of GRK2 using siRNAs reduced viral titers by 10–100 fold, which supports the participation of GRK2 in the entry process of IAV infection. Moreover, the GRK2 inhibitor methyl 5-[2-(5-nitro-2-furyl)vinyl]-2-furoate (**56**, Figure 12) also blocked viral replication. However, its high IC_50_ value against GRK2 makes no definitive kinase intervention pointing to another mechanism of action that is probably associated with compound **56**.

### Abelson Tyrosine Kinases (ABL)

ABL genes encode for two different ABL proteins in humans, ABL1 and ABL2. The first one is involved in the repair of damaged DNA and cell differentiation, whereas ABL2 binds actin and microtubules to enhance cytoskeletal remodelling.^158^ A drug repurposing campaign of FDA-approved medicines and compounds in advanced clinical development (a total of 290 molecules) was carried out to search for antiviral agents for the treatment of MERS-CoV and SARS-CoV.^159^ An ELISA screening was carried out in the case of MERS-CoV, and a cytopathic effect inhibition assay was used instead for SARS-CoV. Data revealed a total of 66 active hits, from which three were reported to be ABL1 inhibitors. While imatinib (**1**) mesylate and dasatinib (**5**) are active against both infections, nilotinib (**17**) only inhibits SARS-CoV (Figure 12). These three compounds exert their antiviral activity with low toxicity and with EC_50_ values ranging from 2.1 to 17.6 μM. The researchers did not perform any additional experiments in order to disclose mechanistic insights of the infection. Recently, *in vitro* inhibition of SARS-CoV-2 by imatinib (**1**) has been reported with an EC_50_ value of 9.82 μM in Vero E6 cell cultures.^160^ However, based in the previous inhibition of imatinib (**1**) in human coronaviruses, clinical trials for COVID-19 with this ABL inhibitor have started (NCT04394416) despite the low possibility of reaching a high enough plasma or lung concentration in human with the standard imatinib dosage (400 or 800 mg/day).^161^ Moreover, recent preclinical studies using the golden Syrian hamster model show that imatinib (**1**) fails to prevent SARS-CoV-2 *in vivo* replication despite high drug concentrations in plasma and in the lung.^162^

On the basis of a previous study aimed at the identification of molecules with anti-DENV activity,^108^ Yang and co-workers identified the allosteric inhibitor GNF-2 (**57**, Figure 12 and Table 1) which inhibited ABL (IC_50_ = 138 nM) and showed a reduction in DENV activity affecting both the viral entry and replication steps.^163^ Compound **57** was proven to target the DENV E protein extracellularly and ABL inside the cell. A structure–activity relationship (SAR) was carried out around **57** in order to obtain selectivity between the ABL-dependent and -independent inhibition pathways. While 4,6-disubstituted pyrimidine analogues were more selective toward ABL, the 2,4-disubstituted counterparts showed more potency against DENV E protein. Although the latter is the most potent inhibitor of the DENV infection (IC_50_ = 5–25 μM), ABL kinase was confirmed to be a valuable therapeutic target for the treatment of DENV infection.

### Calcium/Calmodulin-Dependent Protein Kinases II (CaMKII)

The CaMKII family comprises four highly homologous isoforms (α, β, γ, and δ). These are extensively expressed in brain cells (>1% of the total protein), and they mediate cellular Ca^2+^ to control neuronal plasticity and cognitive functions.^164^ Inhibition of CaMKII has traditionally been a used as a therapeutic strategy for the treatment of heart diseases.^165^ However, CaMKII inhibitors have very recently been proposed to inhibit flavivirus infections such as DENV and ZIKV.^166^ On the basis of previous investigations, a series of benzenesulfonamides were designed and prepared to carry out a SAR study. The synthesized compounds were assessed for their anti-DENV activity using a reporter virus expressing the enhanced green fluorescent protein to infect human neuroblastoma BE(2)C cells. Data revealed that the *N*-phenyl substituent seemed to be crucial for the activity, cycloalkyl groups in the “terminal part” improved activity, and the benzenesulfonamide ring tolerates few modifications. The best CaMKII inhibitor (BSA9, **58**, IC_50_ = 0.79 μM, Figure 12 and Table 1) was able to inhibit infection with both DENV and ZIKV (EC_50_ = 1.52 and 1.91 μM, respectively) in BE(2)C cells with no effects on cytotoxicity, cell proliferation, or cell viability. Regarding the mechanism of action, time-of-addition experiments showed that **58** blocks cellular entry in a concentration-dependent manner. Importantly, the optimized compound increased survival in DENV- and ZIKV-infected mice, suggesting inhibition of CaMKII as a novel therapeutic strategy for the treatment of these types of infections.

### Ataxia Telangiectasia and Rad3-Related Kinase (ATR)

ATR kinase is an important protein for ensuring faithful DNA replication during the S phase of the cell cycle.^167^ Very recently, inhibition of ATR has been proposed as a novel strategy for treatment in the COVID-19 pandemic, caused by SARS-CoV-2.^39^ They used a library of 430 reported PKIs to identify the nucleoside analogue berzosertib (**59**, Figure 12), a selective ATR kinase inhibitor, which showed remarkable antiviral activity in Vero-E6 cells and in HEK293T cells, overexpressing the angiotensin converting enzyme 2 (ACE2) receptor. Regarding the mechanism of action, **59** is known to inhibit the DNA damage response pathway,^168^ and they observed an activation of this pathway upon SARS-CoV-2 infection in Vero-E6 cells. However, further experiments should be carried out in order to validate this hypothesis.

### Adenosine 5′ Monophosphate-Activated Protein Kinase (AMPK)

AMPK is known to be a critical sensor for the regulation of metabolism,^169^ and some studies have linked its deregulation with viral replication.^170^ An interesting example published in 2013 stated that AMPK was required for EBOV entry, so its inhibition may provide a new avenue for the development of antivirals.^171^ They demonstrated that functional AMPK was necessary for EBOV infection, since it was drastically reduced in mouse embryonic fibroblasts lacking the catalytic subunits of the kinase. Besides, the selective inhibitor dorsomorphin (**60**, Figure 12 and Table 1) was able to block EBOV replication in Vero cells in a dose-dependent manner; it was most effective at early times of addition. Importantly, dorsomorphin was effective in decreasing the infection of human macrophages, which are critical targets of EBOV *in vivo*.

### Phosphatidylinositol 3-Kinases (PI3K)/Akt/Mammalian Target of the Rapamycin (mTOR) Pathway

Another central lipid kinase is phosphatidylinositol 3-kinase (PI3K), which phosphorylates inositol phospholipids to produce PtdIns3P, phosphatidylinositol-3,5-biphosphate (PtdIns(3,5)P_2_), and phosphatidylinositol-3,4,5-triphosphate (PtdIns(3,4,5)P_3_).^172^ This kinase and its downstream effectors, Akt (also known as protein kinase B) and mTOR activates many intracellular signaling pathways that regulate diverse functions such as vesicle trafficking, cell metabolism or survival.^173^ Regarding their structure and substrate specificity, PI3Ks can be divided into three classes (I, II, and III), where class I has been the most studied.^174^ Upregulation of PI3Ks has been traditionally linked to the development of human cancer. In fact, there are a great number of ongoing clinical trials with PI3K inhibitors for the treatment of different types of cancer, and three compounds have received FDA approval for commercialization (idelalisib, copanlisib, duvelisib).^175^

There is also scientific evidence that PI3K is involved in different phases of the viral infection process, such as viral entry, viral genome replication, and its translation into proteins.^176^ Particularly, there have been extensive studies regarding the activation of PI3K linked to viral uptake. For example, IAV attachment to the membrane of A549 lung epithelial cells induces the creation of lipid clusters which activate the PI3K/Akt signaling pathway, promoting viral internalization.^79^ Infection of Vero-E6 cells with Zaire EBOV is impaired by preventing its entry using specific inhibitors of PI3K (LY294002 (**61**), Figure 12) and its downstream effector Atk (no specified).^177^ Additionally, inhibitor **61** has also been used to block the entry of other viruses such as HSV-1^178^ and African swine fever virus.^179^ In contrast to most studies, a recent article states that inhibition of PI3K with **61** increased viral titers in several cell lines when infected with West Nile virus (belonging to the *Flaviviridae* family), provoking also a downregulation of the IFN-1 signaling pathway.^180^ However, the molecular mechanisms that link PI3K activation with viral infection remain elusive in most cases, and more investigation needs to be carried out to shed light on this area.

### Sphingosine Kinase (SphK)

Sphingolipids are molecules involved in signal transduction inside and outside the cell. The phosphorylation of sphingosine to its active monophosphate form (S1P) is catalyzed by the different SphK isozymes (SphK1 and SphK2). Inhibitors of SphK have been shown to have a place in reduction SARS-CoV-2 replication and viral load.^181^ Opaganib (**19**, Figure 12), a specific SphK2 inhibitor with a *K*_i_ of 9.8 μM,^182^ is able to reduce the inflammatory pathway, and it simultaneously reduces viral propagation in an *in vitro* model of human lung bronchial tissue.^183^ On the basis of these properties, compound **19**, a lipid kinase inhibitor, was evaluated in clinical trials phase 2/3 for severe COVID-19 patients requiring hospitalization and oxygen treatment (NCT04467840) with good results.

## 9 Concluding Remarks

Kinases are involved in a myriad of physiological events, that are considered bona fide druggable targets. To date, kinase inhibitors are in clinical use for the treatment of cancer and inflammatory processes. However, because of the therapeutic potential of kinases, the scope of diseases is growing faster and is being expanded significantly beyond nononcologic diseases.^184^ In this perspective, we have revisited the state-of-art of kinase inhibitors, PKIs and LKIs, as alternative drugs for the treatment of viral infections based on host targets.

Available kinase inhibitors approved by the FDA or in advance regulatory phases offer a unique opportunity to go deeper into their role in other diseases. However, since FDA-approved medicines and compounds in advanced clinical trials have already gone through an extensive optimization, repurposing strategies usually do not provide much improvement. Moreover, drug repurposing programs may help gain a better understanding on how kinases function during a viral infection and, together with all the structural knowledge already available, could assist the beginning of successful medicinal chemistry programs both in academia and industry, which are currently very scarce, and render new hits that could give more flexibility to improving pharmacokinetic and biological properties.

A great body of evidence supports the relationship between PK inhibition and antiviral properties. Examples of such data encompass important PK families such as NAKs, RTKs, MAPKs, Src kinases, and CDKs, among others. As a result, there have been some successful results within drug repurposing campaigns with the identification of old compounds with antiviral properties as a novel strategy for the treatment of viral infections. Notably, important results have been obtained with the multikinase inhibitor dasatinib (**5**), which has shown remarkable potential as an antiviral agent both alone and in combination with other compounds. Nevertheless, there are still many aspects to be discerned in the use of dasatinib (**5**) regarding the mechanism of action or toxicity issues.

In the case of LKs, regardless of their critical role in a variety of cellular functions, inhibitors have been identified that are able to reach different phases of clinical trials, but none of them have been yet commercialized by the FDA. Probably, further experimentation needs to be carried out in this field to fully discern and understand the mechanism of action of this type of inhibitors. PIKfyve and PI3K are promising LKs that have shown a great potential for the development of broad-spectrum antivirals.

In summary, extensive use of FDA-approved kinase inhibitors has been quite useful for deciphering the role of host kinases in viral infection. While specific kinase inhibitors are needed to completely unveil their mechanism of action, intensive medicinal chemistry programs focused on the optimization of kinase inhibitors with antiviral properties are also required. This is one of the challenges to be faced by scientists in this field in order to obtain high-quality kinase inhibitors as future antiviral drugs.

## Abbreviations

AAK1: adaptor-associated kinase 1
Ab: antibody
ABL: Abelson tyrosine kinase
ACE2: angiotensin converting enzyme 2
AGC: protein kinase A, G, and C families
Akt: protein kinase B
ALS: amyotrophic lateral sclerosis
AMPK: adenosine-5′-monophosphate-activated protein kinase
AP: adaptor protein
AP-1: activator protein 1
AP2: adaptor protein 2
AP2M1: μ subunit of the AP2 complex
ASFV: African swine fever virus
ATP: adenosine triphosphate
ATR: ataxia telangiectasia and Rad3-related kinase
BIKE/BMP2K: BMP-2 inducible kinase
CaMKII: calcium/calmodulin-dependent protein kinases II
CaMKs: Ca^2+^/calmodulin-dependent protein kinases
CDKs: cyclin-dependent kinases
CK: casein kinase
CLK: CDC-like kinase
COVID-19: coronavirus disease 2019
DAAs: direct-acting antivirals
DENV: dengue virus
DNA: deoxyribonucleic acid
EBOV: Ebola virus
EGF: epidermal growth factor
EGFR: epidermal growth factor receptor
ELISA: enzyme-linked immunosorbent assay
ERKs: extracellular signal-regulated kinases
FDA: Food and Drug Administration
GAK: cycling G-associated kinase
GF: growth factor
GFR: growth factor receptor
GPCR: G protein-coupled receptor
GRKs: G protein-coupled receptor kinases
GSK: glycogen synthase kinase
HAdV: human adenovirus
HCMV: human cytomegalovirus
HCV: hepatitis C virus
HCVcc: cell culture HCV particles
HIV-1: human immunodeficiency virus-1
HSV-1: herpes simplex virus type 1
HSV-2: herpes simplex virus type 2
HTS: high-throughput screening
IAV: influenza A virus
IBV: influenza B virus
IC_50_: half maximal inhibitory concentration
IFN-1: type 1 interferon
IPEC: porcine intestinal columnar epithelial cells
JEV: Japanese encephalitis virus
JNK: janus kinase
LKI: lipid kinase inhibitor
LK: lipid kinase
MAPKs: mitogen-activated protein kinases
MAPKAPK: MAPK-activated protein kinases
MAPKKs: MAPK kinases
MAPKKKs: MAPK kinase kinases
MDCK: Madin-Darby canine kidney
MERS-CoV: middle east respiratory syndrome coronavirus
MEK: MAPK/ERK kinase
MOI: multiplicity of infection
mTOR: mammalian target of rapamycin
NAK: Numb-associated kinase
NFAT: nuclear factor of activated T-cells
NF-κB: nuclear factor κ-light-chain-enhancer of activated B cells
NPC1: Niemann-Pick C1 receptor
PBMC: peripheral blood lymphocytes
PDB: Protein Data Bank
PDGFR: platelet-derived growth factor receptor
PHH: primary human hepatocyte
PI3K: phosphatidylinositol-3-kinase
PIKfyve: phosphatidylinositol-3-phosphate-5 kinase
PKC: protein kinase C
PKI: protein kinase inhibitor
PKs: protein kinase
PtdIns: phosphatidylinositol
PtdIns(3,5)P_2_: phosphatidylinositol-3,5-biphosphate
PtdIns3P: phosphatidylinositol-3-phosphate
RAF: rapidly accelerated fibrosarcoma
RGCs: receptor guanylate cyclases
RNA: ribonucleic acid
RNP: ribonucleoprotein
RTK: receptor tyrosine kinase
SAMHD1: SAM domain and HD domain 1
SAR: structure–activity relationship
SARS-CoV: severe acute respiratory syndrome coronavirus
SARS-CoV-2: severe acute respiratory syndrome coronavirus 2
SILAC: stable isotope labeling with amino acids in cell culture
siRNA: small interfering RNA
STE: Ser/Thr kinase
STK16: Ser/Thr kinase 16
TAM: Tyro3 Axl Mer
TGEV: transmissible gastroenteritis virus
TGF-α: transforming growth factor-α
TK: tyrosine kinase
TKL: tyrosine kinase-like kinase
TrKA/HER2: tropomyosin receptor kinase A/human epidermal growth factor receptor 2
vRNP: viral RNP
ZIKV: ZIKA virus
